# Cardiac magnetic resonance feature tracking myocardial strain analysis in suspected acute myocarditis: diagnostic value and association with severity of myocardial injury

**DOI:** 10.1186/s12872-023-03201-2

**Published:** 2023-03-28

**Authors:** Qian Gao, Wenfang Yi, Chao Gao, Tianfu Qi, Lili Li, Kaipeng Xie, Wei Zhao, Wei Chen

**Affiliations:** Department of Radiology, The First Affiliated Hospital of Kunming Medical University, 295, Xichang Road, Wuhua District, Kunming, 650032 Yunnan Province Republic of China

**Keywords:** Myocarditis, Cardiac magnetic resonance imaging, Feature tracking, Strain, Updated Lake Louise Criteria

## Abstract

**Background:**

Albeit that cardiac magnetic resonance feature tracking (CMR-FT) has enabled quantitative assessment of global myocardial strain in the diagnosis of suspected acute myocarditis, the cardiac segmental dysfunction remains understudied. The aim of the present study was using CMR-FT to assess the global and segmental dysfunction of the myocardium for diagnosis of suspected acute myocarditis.

**Methods:**

Forty-seven patients with suspected acute myocarditis (divided into impaired and preserved left ventricular ejection fraction [LVEF] groups) and 39 healthy controls (HCs) were studied. A total of 752 segments were divided into three subgroups, including segments with non-involvement (S_Ni_), segments with edema (S_E_), and segments with both edema and late gadolinium enhancement (S_E+LGE_). 272 healthy segments served as the control group (S_HCs_).

**Results:**

Compared with HCs, patients with preserved LVEF showed impaired global circumferential strain (GCS) and global longitudinal strain (GLS). Segmental strain analysis showed that the peak radial strain (PRS), peak circumferential strain (PCS), and peak longitudinal strain (PLS) values significantly reduced in S_E+LGE_ compared with S_HCs_, S_Ni_, S_E_. PCS significantly reduced in S_Ni_ (-15.3 ± 5.8% vs. -20.3 ± 6.4%, *p* < 0.001) and S_E_ (-15.2 ± 5.6% vs. -20.3 ± 6.4%, *p* < 0.001), compared with S_HCs_. The area under the curve (AUC) values of GLS (0.723) and GCS (0.710) were higher than that of global peak radial strain (0.657) in the diagnosis of acute myocarditis, but the difference was not statistically significant. Adding the Lake Louise Criteria to the model resulted in a further increase in diagnostic performance.

**Conclusions:**

Global and segmental myocardial strain were impaired in patients with suspected acute myocarditis, even in the edema or relatively non-involved regions. CMR-FT may serve as an incremental tool for assessment of cardiac dysfunction and provide important additional imaging-evidence for distinguishing the different severity of myocardial injury in myocarditis.

**Supplementary Information:**

The online version contains supplementary material available at 10.1186/s12872-023-03201-2.

## Background

As an important cause of cardiac morbidity and mortality, myocarditis has been reported in up to 12% of young adults with sudden death [1, 2] and regarded as an important etiology underlying some other myocardial diseases [3]. However, its diagnosis remains challenging and formidable due to its diversified clinical presentation. Endomyocardial biopsy, the current gold standard of myocarditis, is unsatisfactory due to its low sensitivity and specificity and invasiveness [4, 5]. Cardiac magnetic resonance (CMR), combining morphological and functional imaging with myocardial tissue characterization, has enabled the detection of myocardial tissue characteristic of acute inflammation (e.g., cardiac dysfunction, myocardial edema, hyperemia, and necrosis), and is thus increasingly employed for diagnostic evaluation of patients with suspected acute myocarditis [6, 7]. The current widely-accepted CMR-based criteria of myocarditis, the updated Lake Louise Criteria (LLC), relies on at least one T2-based criterion (increased myocardial T2-mapping or signal intensity in T2-weighted images) and one T1-based criterion (increased myocardial T1-mapping, extracellular volume (ECV), or late gadolinium enhancement (LGE)) [8]. Recently, cardiac magnetic resonance myocardial feature tracking (CMR-FT) has been introduced as a new method for high-resolution assessment of global and regional myocardial deformation by tracking the actual myocardial borders and following them over time [9, 10]. This technique allows accurate evaluation of circumferential, radial, and longitudinal myocardial strain and has been already applied in a wide range of cardiovascular conditions [10]. Albeit a few recent studies of the feasibility, sensitivity, and specificity of global myocardial strain parameters based on CMR-FT in the diagnosis of myocarditis [6, 11–14], there has been scant studies focusing on the segmental dysfunction of the myocardium by using CMR -FT. Recently, the 2D speckle tracking echocardiography (2D-STE) has been successfully employed to quantify myocardial deformations, which may be differentially manifested in suspected acute myocarditis [15]. It is worth noting, nevertheless, the 2D-STE technique has some intrinsic limitations of its own. The most critical one lies in its temporal instability of tracking patterns [16]. However, there has been a lack of CMR-FT studies related to edema or necrosis based on T2-weigehted and LGE images to quantitatively evaluate cardiac segmental dysfunction in suspected acute myocarditis. It remains to be clarified whether the severity of myocardial injury (e.g. edema, necrosis) is related to the regional myocardial dysfunction. Besides, it is also necessary to further probe into the diagnostic value of different strain parameters for patients with suspected acute myocarditis, especially in preserved LVEF. It is against this background of niches in this field that the present study has been carried out to look into the global and segmental dysfunction of the myocardium in suspected acute myocarditis and the diagnostic value of CMR-FT myocardial strain analysis. To be specific, this study investigated (1) whether FT-derived strain parameters can be used to differentiate patients with suspected acute myocarditis from healthy controls (HCs), (2) to what extent the changes in segmental strain parameters depend on the severity of myocardial injury in patients with suspected acute myocarditis, and (3) which myocardial strain parameters perform the best in detecting suspected acute myocarditis. We present the following article in accordance with the STARD reporting checklist.

## Methods

### Study population

This retrospective study was approved by the Institutional Review Boards of our hospital (No.2022-L-128). Forty-seven patients (35 males, 12 females) were retrospectively enrolled from 2015 to May 2019 in our hospital. All the patients had been clinically suspected acute myocarditis (mean symptom duration before referral: 5.2 ± 4.1 days, < 14 days for all the patients) and underwent CMR examination. They were diagnosed with myocarditis for having clinical symptoms and according to the corresponding clinical criteria based on 2013 ESC guidelines [17]. Clinically suspected myocarditis refers to symptomatic patients (chest pain, dyspnea, palpitation, or other relevant cardiac symptoms) based on one or more diagnostic criteria (abnormal electrocardiography, elevated troponin, and functional or structural abnormalities confirmed by echocardiography or MR) and asymptomatic patients based on two or more of the above criteria. Coronary artery disease was ruled out prior to CMR in all patients (Table 1). The control group consisted of 39 age- and gender-matched healthy controls (HCs) (26 males, 13 females), who were selected for their uneventful medical histories, absence of symptoms indicative of cardiovascular dysfunction, lack of abnormalities in electrocardiograms, echocardiography and CMR, and no history of inflammatory diseases.

**Table 1:** Classification of patients with suspected acute myocarditis according to clinical criteria [17]

	**Myocarditis patients (*n* = 47)**
**Clinical symptoms consistent with myocarditis**	
** Acute chest pain**	40
** New-onset(days up to 3 months) or worsening of: dyspnea at rest or exercise/fatigue, with or without left and/or right heart failure signs**	15
** Palpitations /arrhythmia symptoms /syncope/ aborted sudden cardiac death**	8
** Cardiogenic shock**	1
**Diagnostic criteria consistent with myocarditis**	
** ECG / Holter / stress test features**	42
** Elevated TnT/TnI**	29
** Functional and structural abnormalities on cardiac imaging (echo/angio/CMR)**	12
**Exclusion of coronary artery disease (CAD)**	
** Cardiac catheterization**	17
** Cardiac computed tomography angiography**	30

### Image acquisition

CMR was performed on a 3.0-T MR system (Achieva, Philips Healthcare, Best, Netherland) using a standard 16-element cardiac phased array coil and a four-lead vectorcardiogram. For functional analysis, electrocardiography-gated balance turbo field echo (b-TFE) cine images were obtained in the short-axis, four-chamber, three-chamber, and two-chamber views (TR, 3.2 ms; TE, 1.53 ms; FOV, 320 mm × 320 mm; reconstruction matrix, 320 × 320; flip angle, 45°; slice thickness, 8 mm; space, 2 mm). Edema-sensitive black-blood T2-weighted images with fat saturation (triple inversion recovery turbo spin echo sequences with inversion pulses for fat and blood suppression) were acquired in the short-axis orientation covering the entire left ventricle (TR, 2000 ms; TE, 60 ms; FOV, 320 mm × 320 mm; reconstruction matrix, 352 × 352; slice thickness, 8 mm; space, 2 mm). Late gadolinium enhancement (LGE) imaging was performed 10 min after the injection of 0.1 mmol/kg gadobutrol (Gadovist, Bayer Healthcare, Leverkusen, Germany) with inversion time (300–340 mm) adjusted according to a Look-Locker inversion recovery-prepared T1-weighted phase-sensitive inversion recovery sequence (TR, 6.1 ms; TE, 3.0 ms; TI, 400 ms; FOV, 320 mm × 320 mm; reconstruction matrix, 320 × 320; flip angle, 25°; slice thickness, 8 mm; space, 2 mm) in the short-axis, four-chamber, three-chamber, and two-chamber views, which corresponded with cine images. CMR scanning was performed according to the standardized protocols recommended by the Society for Cardiovascular Magnetic Resonance (SCMR) [18].

### Imaging analysis

Two radiologists experienced in CMR diagnosis blindly analyzed the data, performed the measurements, and reached agreement regarding the consequences. Prior to FT analysis, a random cohort of 15 patients was chosen to assess intra- and interobserver reproducibility.

### Cardiac function analysis

Cardiac function analysis was performed offline based on the acquired b-TFE cine images by using dedicated software (CVI 42 v. 5.6, Circle Cardiovascular Imaging Inc., Calgary, AB, Canada). Endocardial and epicardial contours of the left ventricle were manually delineated at the end-systolic and end-diastolic phases in short-axis views to calculate volume changes and left ventricular ejection fraction (LVEF). Based upon the body surface area, LV end-diastolic volume index, LV end-systolic volume index, and myocardial mass index were quantified. Patients with acute myocarditis were divided into two subgroups according to LVEF, including the impaired-LVEF group (LVEF < 55%; *n* = 12) and the preserved-LVEF group (LVEF ≥ 55%; *n* = 35) [11].

### The updated LLC

Image analysis of the updated LLC was performed using the CVI 42 software (CVI 42 v. 5.6, Circle Cardiovascular Imaging Inc., Calgary, AB, Canada). The myocardium was divided into 16 segments according to the American Heart Association segmentation [19]. Every segment was evaluated for the following tissue characterizations: 1) T2-based marker for myocardial edema with either T2-weighted imaging or T2 mapping, and 2) T1-based marker for associated myocardial injury: one of the three methods, namely, LGE, T1-mapping or extracellular volume (ECV).

CMR diagnosis of myocarditis was based on the edema-sensitive CMR (T2-weighted images or T2 mapping) and at least one additional T1-based tissue characterization technique [8]. As T1 and T2 mapping and ECV are not routine sequences in our institution, the approach chosen by our study included only T2-weighted imaging and LGE. T2-weighted imaging is identified visually on T2-weighted black-blood imaging and by calculating the T2 ratio of ≥ 1.9 (signal intensity normalized to skeletal muscle in the same slice) [8, 20]. The patterns of LGE are commonly and typically located in the subepicardial and midmyocardial regions [8]. All the myocardial segments of the enrolled patients were divided into three subgroups based on the severity of their myocardial injury [21], viz. segments with non-involvement (S_Ni_; *n* = 509), segments with edema (S_E_; *n* = 89), and segments with both edema and LGE (S_E+LGE_; *n* = 154). The S_E_ subgroup was localized using T2-weighted images; the S_E+LGE_ subgroup was localized using both T2-weighted images and LGE images; and the S_Ni_ subgroup was considered as normal in comparison with the two preceding subgroups. A cohort of segments of HCs (S_HCs_; *n* = 272) served as the control group.

### Myocardial strain analysis using CMR-FT

**Fig. 1:**
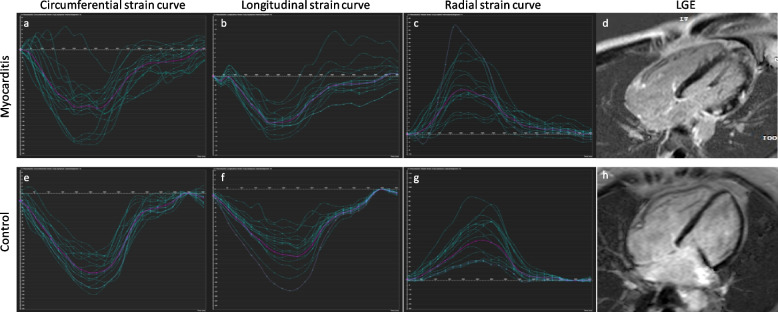
Circumferential strain curves, longitudinal strain curves, radial strain curves, and late gadolinium enhancement images (four-chamber view) for a patient with acute myocarditis (**a**-**d**, 28-year-old male; ejection fraction, 69.4%) and a healthy control (**e**–**h**, 30-year-old female; ejection fraction; 68.6%). The strain graph shows the circumferential, longitudinal, and radial strain of each segment (blue curves) vs. global strain (pink curve). Average circumferential, longitudinal, and radial strain are clearly reduced in the patient with acute myocarditis (global peak circumferential strain: -21.32% vs.-14.74%; global peak longitudinal strain: -13.53% vs. -10.0%; global peak radial strain: 38.67% vs. 22.05%). On late gadolinium enhancement imaging, typical patchy subepicardial and midmyocardial inflammatory/necrotic lesions are visible

CMR-FT was performed offline based on the acquired b-TFE cine images using CVI 42 software (CVI 42 v. 5.6, Circle Cardiovascular Imaging Inc., Calgary, AB, Canada). Endocardial and epicardial contours were drawn manually in the end-diastolic phases, and myocardial strain was automatically tracked by CVI 42 throughout the cardiac cycle. Global peak longitudinal strain (GLS) was averaged from the measurements of the two-, three-, and four-chamber views. Circumferential (GCS) and radial (GRS) peak strain parameters were determined in the short-axis view covering the entire left ventricle (Fig. 1). Every segment was evaluated for myocardial strain parameters to obtain segmental peak radial strain (PRS), peak circumferential strain (PCS), and peak longitudinal strain (PLS).

### Statistical analysis

Normality was tested with the Kolmogorov–Smirnov test. Continuous variables were presented as means ± standard deviations and compared using the Student’s t-test for normally distributed data or the Mann–Whitney U-test for non-normally distributed data. The inter- and intra-observer variability was assessed with the intraclass correlation coefficient (ICC) and Bland–Altman plot. Parametric data of more than two groups were compared using one-way analysis of variance testing. Post-hoc testing was performed with the least significant difference test (homogeneity of variance) or Dunnett’s T3 test (heterogeneity of variance). Categorical group data presented as percentages were compared using the chi-squared test or Fisher’s exact test, as appropriate. Diagnostic performance of the strain parameters was analyzed by plotting receiver operating characteristic curves and comparing the areas under those curves. Cutoff values were defined by calculating the Youden index for the predictive variables, and sensitivity, specificity, and accuracy were calculated. To combine the single predictive variables, scores were derived from logistic regression analysis. The level of statistical significance was set to *p* < 0.050. Statistical analysis was performed using SPSS software (v.25.0, IBM SPSS Statistics, Armonk, NY, USA).

## Results

### Intra- and interobserver agreement

The reproducibility was good for global and segmental stain parameters, with ICC of inter-observer ranges from 0.882 to 0.983 for global parameters and 0.837 to 0.883 for segmental parameters, the ICC of intra-observer ranges from 0.986 to 0.996 for global parameters and 0.877 to 0.977 for segmental parameters. The detailed ICC and 95% CI of inter- and intra-observer agreement for different strain parameters and the Bland–Altman plots are listed in Additional file 1.

### Population characteristics

This study included forty-seven patients with suspected acute myocarditis and 39 HCs. Clinical symptoms of the patients are given in Table 1. Demographic characteristics of the HCs and the patients, LV volumetric parameters, and updated LLC data are presented in Table 2. In the study, patients with suspected acute myocarditis demonstrated regions of edema and LGE primarily in the inferior and lateral walls of the left ventricular myocardium. Specifically, 28 patients (28/47, 59.6%) showed edema and LGE in the inferior wall, 22 patients (22/47, 46.8%) in the lateral wall, 17 patients (17/47, 36.2%) in the interventricular septum, and 9 patients (9/47, 19.1%) in the anterior wall. All the LGE were primarily located in the subepicardial and midmyocardial regions, which was different from myocardial infarction. Meanwhile, 11 (11/39, 28%) healthy controls had scattered LGE in the right ventricle (RV) insertion point or in the septal myocardium, but they did not exhibit any symptoms of cardiovascular dysfunction and showed no abnormalities in ECG, cardiac enzymes, or heart function.

**Table 2:** Characteristics of patients with suspected acute myocarditis (subgroups with preserved and impaired left ventricular ejection fraction) and controls

Parameter	Controls (*N* = 39)	Myocarditis (*N* = 47)	Myocarditis with preserved LVEF (*N* = 35)	Myocarditis with impaired LVEF (*N* = 12)
**Female/male**	13/26	12/35	7/28	5/7
**Age(years)**	33 ± 12	31 ± 13	30 ± 14	32 ± 11
**Height(cm)**	166 ± 8	167 ± 8	168 ± 9	166 ± 8
**Weight(kg)**	60 ± 13	63 ± 12	63 ± 14	61 ± 8
**Heart rate(bpm)**	74 ± 14	74 ± 15	75 ± 17	72 ± 8
**BSA(ml/m**^**2**^**)**	1.64 ± 0.21	1.67 ± 0.20	1.68 ± 0.20	1.64 ± 0.14
**Symptom duration before CMR (days)**	n.a	5.2 ± 4.1	5.0 ± 3.8	5.5 ± 4.7
**Initial TnI (ng/ml, NR:0–0.4)**	n.a	11.1 ± 13.6	10.6 ± 15.1	12.1 ± 10
**Initial MYO (μg/L, NR: < 110)**	n.a	275 ± 298	241 ± 306	279 ± 235
**Initial CK-MB(ng/ml, NR: < 24.0)**	n.a	19.7 ± 20.9	15.6 ± 16.5^c^	33.4 ± 24.5^b^
**Initial CRP(mg/L, NR: < 6.0)**	n.a	60.8 ± 95.7	95.3 ± 110.3^c^	5.6 ± 3.3^b^
**LVED volume/BSA (ml/m**^**2**^**)**	71.6 ± 12.1	80.7 ± 31.2	74.1 ± 16.7^c^	89.6 ± 26.2^ab^
**LVES volume/BSA (ml/m**^**2**^**)**	22.7 ± 6	34.7 ± 26^a^	25.6 ± 7.6^c^	54.9 ± 24.2^ab^
**LV ejection fraction (%)**	68.3 ± 6.2	59.4 ± 14.3^a^	65.5 ± 5.9^c^	40.0 ± 14.8^ab^
**LVED wall mass/BSA(g/m**^**2**^**)(without papillary muscles)**	49.8 ± 12.6	58.1 ± 13.4^a^	54.9 ± 10.1^c^	66.2 ± 17.6^ab^
**T2 Ratio**	1.8 ± 0.2	2.3 ± 0.6^a^	2.2 ± 0.4^a^	2.5 ± 0.9^a^
**2 out of 2 updated LLC (%)**	0	72.3	68.6	83.3
**1 out of 2 updated LLC (%)**	28.2	17.1	17.1	16.7
**0 out of 2 updated LLC (%)**	71.8	10.6	14.3	0

*BSA* Body surface area, *ED* End diastolic, *ES* End systolic, *LV* Left ventricle, *LLC* Lake Louise Criteria, *NR* Normal range

^a^Significant difference compared to controls

^b^Significant difference compared to patients with preserved left ventricular ejection fraction

^c^Significant difference compared to patients with impaired left ventricular ejection fraction

### Global strain analysis between patients and HCs

**Fig. 2:**
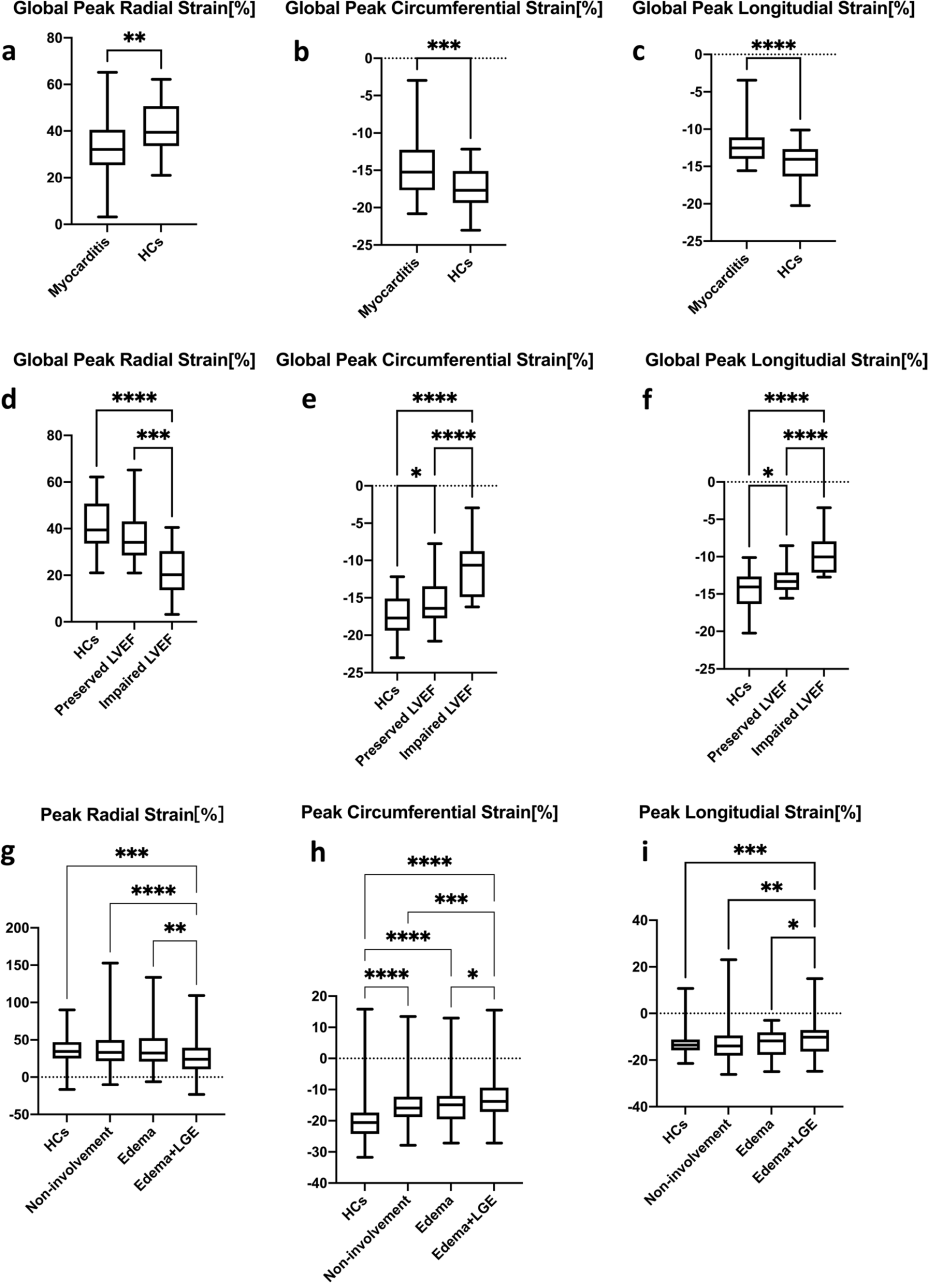
Comparison of global strain parameters between healthy controls and patients with suspected acute myocarditis (**a**-**c**). Comparison of global strain parameters between healthy controls, patients with preserved-LVEF and patients with impaired-LVEF (**d**-**f**). Comparison of segmental strain parameters between healthy controls, non-involvement segments, edema segments, edema and LGE segments (**g**-**i**). *p < 0.05; ** 0.001 < *p* < 0.05; *** 0.0001 < *p* < 0.001; **** *p* < 0.0001

Compared with HCs, patients with suspected acute myocarditis showed significantly impaired GRS (32.5 ± 12.0% vs. 40.8 ± 10.8%; *p* = 0.001), GCS (-14.6 ± 3.7%-vs. 17.4 ± 2.8%; *p* < 0.001), and GLS (-12.2 ± 2.5% vs. -14.5 ± 2.5%; *p* < 0.001) (Fig. 2 a-c). Patients with impaired LVEF showed significantly decreased GRS (21.2 ± 10.3%vs. 40.8 ± 10.8%; *p* < 0.001), GCS (-11.0 ± 3.8%vs. -17.4 ± 2.8%; *p* < 0.001), and GLS (-9.6 ± 2.7% vs. -14.5 ± 2.5%; *p* < 0.001) as compared with HCs (Fig. 2a-c). Patients with preserved LVEF also showed significantly decreased GCS (-15.9 ± 2.8%vs. -17.4 ± 2.8%; *p* = 0.029) and GLS (-13.0 ± 1.8%vs. -14.5 ± 2.5%; *p* = 0.007) as compared with HCs, whereas their GRS values were not significantly different from that of the HCs(36.3 ± 10.1%vs. 40.8 ± 10.8%; *p* = 0.072). The three global strain parameters significantly decreased in patients with impaired-LVEF as compared with that in the preserved-LVEF group (*p* < 0.010) (Fig. 2d-f).

### Segmental strain analysis in patients with suspected myocarditis based on severity of myocardial injury

PRS, PCS, and PLS values significantly reduced in S_E+LGE_ as compared with that in S_HCs_, S_Ni_ and S_E_ groups. PCS significantly reduced in S_Ni_ (-15.3 ± 5.8% vs. -20.3 ± 6.4%, *p* < 0.001) and S_E_ (-15.2 ± 5.6% vs. -20.3 ± 6.4%, *p* < 0.001) groups as compared with that in S_HCs_ group. However, S_Ni_ and S_E_ groups were not significantly different from each other in their PRS, PCS, and PLS (Fig. 2 g-i). All CMR segmental strain parameters for each subgroup evaluated are given in Table 3.

**Table 3:** Segmental strain values of three patient groups and healthy controls

	**Healthy Controls (*n* = 272)**	**Non-involvement segments (*n* = 509)**	**Edema segments (*n* = 89)**	**Edema + LGE segments (*n* = 154)**
**Peak Radial Strain [%]**	36.7 ± 16.1^d^	37.3 ± 23.6^d^	38.5 ± 25.4^d^	28.1 ± 24.3^abc^
**Peak Circumferential Strain [%]**	-20.3 ± 6.4^bcd^	-15.3 ± 5.8^ad^	-15.2 ± 5.6^ad^	-13.0 ± 7.2^abc^
**Peak Longitudinal Strain [%]**	-13.4 ± 3.7^d^	-12.8 ± 7.6^d^	-12.6 ± 5.6^d^	-10.8 ± 7.3^abc^

^a^Significant difference compared to healthy controls

^b^Significant difference compared to non-involvement segments

^c^Significant difference compared to edema segments

^d^Significant difference compared to edema + LGE segments

### Diagnostic performance of strain indices and strain parameters in patients with preserved ejection fraction

**Fig. 3:**
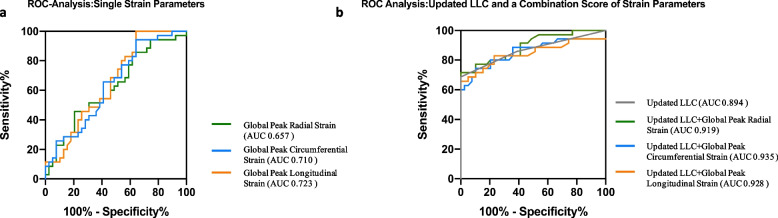
**a** Graph showing receiver operating characteristic curves for global peak longitudinal strain (area under the curve [AUC]: 0.723), global peak circumferential strain (AUC: 0.71), and global peak radial strain (AUC: 0.657) (*p* > 0.05, respectively). **b** Graph showing receiver operating characteristic curves for the updated Lake Louise Criteria (area under the curve [AUC]: 0.894) and for a combination score of global peak longitudinal strain (AUC: 0.928), global peak circumferential strain (AUC: 0.935) (compared with the AUC value of updated LLC, *p* < 0.05, respectively), and global peak radial strain (AUC: 0.919) (compared with the AUC value of updated LLC, *p* = 0.06)

GLS and GCS showed good performance in the diagnosis of suspected acute myocarditis, with area under the curve (AUC) being 0.723 and 0.710, respectively. The diagnostic performance of GRS had an AUC of 0.657 (Fig. 3a). The optimal cutoff values of GRS, GCS, and GLS were 33.08%, -17.07%, and -12.77%, respectively. The updated LLC yielded excellent diagnostic performance (AUC: 0.894). However, the diagnostic performance of the combined scores of GCS, GLS, and GRS with updated LLC were further improved (AUC: 0.935, 0.928, and 0.919, respectively) (Fig. 3b). Sensitivities, specificities, accuracies, positive predictive values, and negative predictive values for all parameters are given in Table 4.

**Table 4:** Diagnostic performance of different cardiac magnetic resonance strain parameters for diagnosis of suspected acute myocarditis

	**AUC**	**Sensitivity [%]**	**Specificity [%]**	**Accuracy [%]**	**PPV [%]**	**NPV [%]**
**Global Peak Radial Strain**	0.657	53	79	65	75	58
**Global Peak Circumferential Strain**	0.710	74	56	66	67	64
**Global Peak Longitudinal Strain**	0.723	60	74	66	74	60
**Combinations**						
** Updated LLC**	0.894	72	100	85	100	75
** Updated LLC + Global Peak Radial Strain**	0.919	77	100	87	100	87
** Updated LLC + Global Peak Circumferential Strain**	0.935	85	90	87	91	83
** Updated LLC + Global Peak Longitudinal Strain**	0.928	79	97	87	97	79

## Discussion

We retrospectively investigated the global and segmental dysfunction of the myocardium and diagnostic value of CMR-FT myocardial strain analysis in suspected acute myocarditis. The major findings of this study are as follows: firstly, cardiac deformations measured by CMR-FT were significantly impaired in patients with suspected acute myocarditis and even in those with preserved LVEF; secondly, compared with global analysis, analysis of segmental myocardial strain made it possible to distinguish myocardial injuries in more detail. PRS, PLS and PCS were significantly impaired in the edema and LGE/necrosis segments. PCS was significantly reduced in the LGE negative (edema or non-involvement segments) myocardium in suspected acute myocarditis. Lastly, GLS and GCS derived from CMR-FT showed good performance in diagnosis of patients with suspected acute myocarditis.

### Differences in myocardial strain parameters

Strain parameters like GLS and GCS were considerably reduced in patients with suspected acute myocarditis and even in those with preserved LVEF as compared with that in HCs. However, there was no difference in GRS between patients with preserved LVEF and HCs, which is in agreement with the results of previous CMR and echocardiographic studies [11–14, 22–24]. Overall, these results suggest that myocardial strain parameters, esp. longitudinal and circumferential strain parameters, can detect even subtle alterations in myocardial function [13, 14]. There are probably two reasons for this. Firstly, the myocardium of the left ventricle typically consists of three myocardial layers, including the inner oblique, the middle circular, and the outer oblique. Fibers on the middle and outer myocardial layers produce both circumferential and longitudinal deformation during systole [25, 26]. However, the radial deformation is determined by the three layers of the myocardium. Edema and necrotic inflammatory changes in acute myocarditis most commonly affect the subepicardial layer of the myocardium. During systole, the inner oblique layer undergoes the biggest dimensional changes whereas functional alterations predominantly occur only when the endocardial layer is involved [27]. Therefore, circumferential and longitudinal function might be affected early even if LVEF is preserved. In comparison, radial function might be less affected in myocarditis, which mostly does not affect the endocardium. Radial function might be damaged when ejection fraction is impaired, which may indicate that the endocardial layer is affected. Secondly, previous CMR-FT studies reported that global radial strain showed considerably lower intra- and inter-observer reproducibility as compared with longitudinal and circumferential strain. Some researchers [9, 28] proposed that this may be related to the measurement of an interaction between the endocardial border and the myocardial border during CMR feature tracking, which is not necessary for the derivation of longitudinal and circumferential strain parameters. As the distance between the endocardium and the epicardium is small, there may be systolic elimination of visible blood spaces between trabeculae that exaggerate the apparent shift of the endocardial boundary. To be brief, CMR-FT can detect cardiac systolic dysfunction of myocarditis earlier than the traditional ejection fraction, and GLS and GCS can be effective indicators for early detection of cardiac dysfunction of myocarditis.

### Severity of myocardial injury and segmental myocardial strain parameters

As it is known that different myocardial segments are not equally affected by inflammatory processes in acute myocarditis. Histopathological features of acute myocarditis include cellular infiltration, edema, necrosis, and fibrotic scars [29, 30]. Compared to global analysis, segmental myocardial strain analysis makes it possible to distinguish the myocardial injury in more detail. Recently, Chen et al. [31] has found that even the values of GRS, GCS and GLS were significantly lower in the LGE negative (with or without edema) group than that in the control group. Impairment of the myocardial strain was more severe in patients with LGE positive myocarditis. Ravesh et al. [32] found that the segmental myocardial strain (longitudinal, radial, and circumferential) made it possible to differentiate patient groups with different EF values, and they also found that the circumferential deformation analysis was more sensitive for patients with inconspicuous EF values. However, there is limited data in the literature on the correlation between severity of myocardial injury and segmental myocardial strain parameters. The current study found that segmental PLS, PCS, and PRS values were markedly impaired in the edema and LGE positive segments, thus indicating that myocardial injury is more severe in the presence of LGE. These findings are consistent with that by previous investigations [31, 33]. Strikingly, even reduced PCS values in edema–only and non-involved segments can be detected as compared with healthy control segments. However, PLS and PRS values of non-involved, edema and healthy segments only marginally changed. This study helps confirm the proposition that the edema, or even relatively normal myocardium, suffers from significant contractile impairment early after myocarditis. These phenomena may be ascribed to three causes. Firstly, histopathological analysis suggested that the myocardial edema reduces its contractility by enlarging the distance between actin and myosin filaments [34]. Secondly, unlike myocardial infarction which is typically related to coronary artery supply territories [35], the non-involvement and edema segments are patchily distributed in myocarditis [36], suggesting that the non-involvement segments may be impacted by the adjacent edema region, which may explain the reduced contractility of the non-involvement segments. Lastly, the circumferential strain may be the most sensitive parameter for regional deformation as compared with longitudinal and radial deformation. These segmental strain parameters could reflect early changes in disease and might help to improve diagnostic accuracy of conventional CMR imaging in suspected acute myocarditis, even in the absence of LGE.

In recent years, given the highly variable natural history and prognosis of myocarditis (ranging from complete recovery to severe cardiomyopathy or sudden death), there is great interest in techniques for predicting the risk of future adverse outcomes. LGE and abnormalities of GLS have been shown to be powerful predictors of adverse events in myocarditis [23, 30, 37–40]. Meanwhile, in previous studies, improvement in regional contractile function was found after the regression of edema in acute myocardial infarction [35]. This study finds the LGE negative segments have preserved longitudinal strain in acute myocarditis, suggesting that the LGE negative myocardium may have a lower incidence of adverse events, and thus may recover after edema regression. This finding may provide new evidence for risk stratification of myocarditis, which may help improve patient care [41], and shed some light on the mechanism underlying the prognostic value of myocardial edema imaging.

### Diagnostic performance of FT-derived strain indices

The current CMR criteria (updated LLC) for diagnosis of myocarditis use tissue characteristics. The evaluation of cardiac function has only supportive character in the updated LLC. However, as many myocarditis patients show impaired cardiac strains despite their preserved LVEF, the assessment of cardiac deformation has a potential to tap for improving diagnosis of myocarditis of these patients. The current study was intended to investigate the diagnostic performance of strain parameters of the preserved-LVEF group. Although GLS and GCS showed moderate diagnostic potential (AUC: 0.723 and 0.71, respectively) for suspected acute myocarditis, they also revealed a considerable overlap between HCs and patients with suspected myocarditis. GRS presented a slightly lower diagnostic performance (AUC: 0.693), which supports previous studies [13, 14]. We focused only on suspected myocarditis patients with preserved LVEF, hoping that we might help explain the lower diagnostic performance of strain parameters as compared with a recent study by Luetkens et al. [6], who demonstrated a considerably better diagnostic performance of GLS alone (AUC: 0.83), possibly because patients in their study were not divided into subgroups based on whether LVEF was preserved or not. In our study, the updated LLC still exhibited a high diagnostic potential (AUC: 0.893). The combination of updated LLC with strain parameters may outperform the updated LLC in our study. Thus, we suggest that GCS and GLS may serve as novel parameters for detecting myocardial dysfunction in patients with suspected acute myocarditis and even in those with preserved LVEF. Therefore, strain assessment may have some incremental diagnostic advantages over traditional CMR imaging features.

### Limitations

The present study has several limitations of its own. Firstly, we used a clinical reference standard for suspected myocarditis patients as subjects without endomyocardial biopsy. However, we carefully defined the patient cohort based on clinical criteria similar to that of other studies [6, 7]. Nevertheless, some patients, in whom a differential cause of disease might have been missed, may possibly have some confounding effect on our cohort. Secondly, the number of patients was relatively small, which may affect the generalization of the research. Therefore, in future larger and prospective studies, sub-analyses should be performed with respect to different presentation types to determine the additional value of strain analysis in different clinical scenarios. Despite that CMR offers the possibility of advanced tissue characterization by using mapping technique [13, 14], the assessment of these techniques was beyond the scope of this study. Thus, further investigation by combining cardiac strain analysis with tissue mapping techniques is needed for future research.

## Conclusions

Global and segmental myocardial strain were impaired in patients with suspected acute myocarditis, even in the edema or relatively non-involved regions. CMR-FT may serve as an incremental tool for assessment of cardiac dysfunction and provide important additional imaging-evidence for distinguishing the different severity of myocardial injury in myocarditis.

## Supplementary Information

**Additional file 1:** **Supplementary Table. **Intra-observer and inter-observer variability of different strain parameters. **Appendix Fig.** Bland-Altman plot of intra-(a) and inter-observer(b) reproducibility, for three segmental strain types. The horizontal green line depicts the mean; the 2 red lines depict the upper and lower 95% limit of agreement. PRS, segmental radial strain; PCS, segmental circumferential strain; PLS, segmental longitudinal strain.

## Data Availability

The datasets used and analysed during the current study are available from the corresponding author on reasonable request.
