# Supplementary material for: Cardiac magnetic resonance feature tracking myocardial strain analysis in suspected acute myocarditis: diagnostic value and association with severity of myocardial injury
Source: BMC Cardiovasc Disord. 2023 Mar 28;23:162. doi: 10.1186/s12872-023-03201-2 (PMC10053471; doi:10.1186/s12872-023-03201-2)
Supplement: Supplementary file 1 — Additional file 1: Supplementary Table. Intra-observer and inter-observer variability of different strain parameters. Appendix Fig. Bland-Altman plot of intra-(a) and inter-observer(b) reproducibility, for three segmental strain types. The horizontal green line depicts the mean; the 2 red lines depict the upper and lower 95% limit of agreement. PRS, segmental radial strain; PCS, segmental circumferential strain; PLS, segmental longitudinal strain. [file 12872_2023_3201_MOESM1_ESM.docx]

**Supplementary Table** Intra-observer and inter-observer variability of different strain parameters

| Strain | Variability | ICC | 95% CI |
| --- | --- | --- | --- |
| GRS | Intra-observer | 0.995 | 0.984-0.998 |
|  | Inter-observer | 0.973 | 0.920-0.991 |
| GCS | Intra-observer | 0.996 | 0.989-0.999 |
|  | Inter-observer | 0.983 | 0.949-0.994 |
| GLS | Intra-observer | 0.986 | 0.960-0.995 |
|  | Inter-observer | 0.882 | 0.649-0.960 |
| PRS | Intra-observer | 0.966 | 0.957-0.974 |
|  | Inter-observer | 0.859 | 0.818-0.891 |
| PCS | Intra-observer | 0.977 | 0.970-0.982 |
|  | Inter-observer | 0.883 | 0.849-0.909 |
| PLS | Intra-observer | 0.877 | 0.844-0.904 |
|  | Inter-observer | 0.837 | 0.788-0.874 |

GRS, global peak radial strain; GCS, global peak circumferential strain; GLS, global peak longitudinal strain; PRS, segmental peak radial strain; PCS, segmental peak circumferential strain; PLS, segmental peak longitudinal strain; ICC, intraclass correlation coefficient; CI, confidence interval


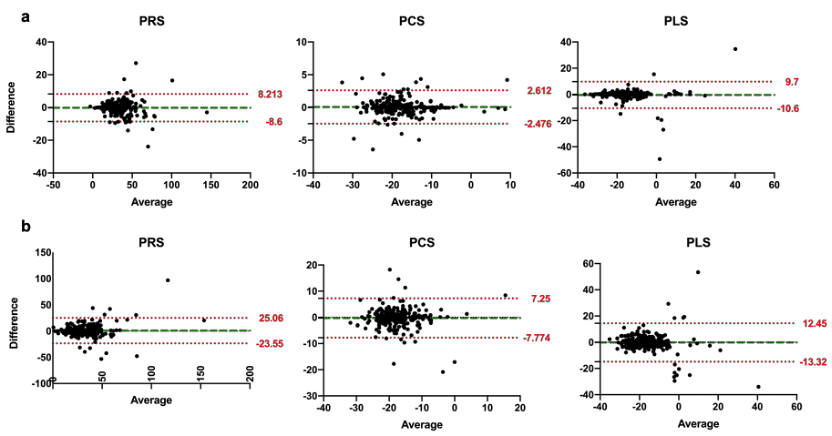


Appendix Fig. Bland-Altman plot of intra-(a) and inter-observer(b) reproducibility, for three segmental strain types. The horizontal green line depicts the mean; the 2 red lines depict the upper and lower 95% limit of agreement. PRS, segmental radial strain; PCS, segmental circumferential strain; PLS, segmental longitudinal strain
